# Espasticidad en Esclerosis Múltiple: Recomendaciones para su Evaluación y Manejo Clínico en 2026

**DOI:** 10.31083/RN51172

**Published:** 2026-10-27

**Authors:** Celia Oreja-Guevara, José Manuel García Domínguez, Paloma Montero Escribano, Lucienne Costa-Frossard, María Luisa Martínez Ginés, Miguel Ángel Llaneza González, Jose María Prieto González, Cristina Ramo-Tello, Ana María Alonso Torres, Carmen Calles, Francisco Pérez Miralles, Sara Eichau, Tamara Castillo-Triviño, Ana Belén Caminero

**Affiliations:** ^1^Servicio de Neurología, Hospital Clínico San Carlos/IdISSC, 28040 Madrid, España; ^2^Departamento de Medicina, Facultad de Medicina, Universidad Complutense de Madrid (UCM), 28040 Madrid, España; ^3^Servicio de Neurología, Hospital General Universitario Gregorio Marañón, 28007 Madrid, España; ^4^HM Hospitales, Servicio de Neurología, 28015 Madrid, España; ^5^Red de Enfermedades Inflamatorias (REI), Madrid, España; ^6^Hospital Ramón y Cajal, Servicio de Neurología, 28034 Madrid, España; ^7^Hospital Universitario Central de Asturias, Servicio de Neurología, 33011 Asturias, España; ^8^Hospital Clínico Universitario de Santiago de Compostela, Servicio de Neurología, 15706 Santiago de Compostela, España; ^9^Instituto de Investigación Sanitaria de Santiago de Compostela (IDIS), 15706 Santiago de Compostela, España; ^10^Hospital Universitario Germans Trias i Pujol, Servicio de Neurología, 08916 Barcelona, España; ^11^Hospital Universitario Regional de Málaga, Servicio de Neurología, 29010 Málaga, España; ^12^Hospital Universitari Son Espases, Servicio de Neurología, 07120 Palma, España; ^13^Hospital Universitario La Fe, Servicio de Neurología, 46026 Valencia, España; ^14^Complejo Hospitalario Virgen Macarena, Servicio de Neurología, 41009 Sevilla, España; ^15^Instituto de Biomedicina de Sevilla, IBiS/CSIC/Universidad de Sevilla, 41013 Sevilla, España; ^16^Servicio de Neurología, Hospital Universitario Donostia, IIS Biogipuzkoa, Neurociencias, 20014 Donostia-San Sebastián, España; ^17^Complejo Asistencial de Ávila, Sección de Neurología, 05071 Ávila, España

**Keywords:** esclerosis múltiple, *spasticity-plus syndrome*, consenso, polifarmacia, multiple sclerosis, spasticity-plus syndrome, consensus, polypharmacy

## Abstract

**Introducción:**

La esclerosis múltiple (EM) es una enfermedad neuroinflamatoria crónica que afecta significativamente a la calidad de vida. La espasticidad es uno de los trastornos más frecuentes e incapacitantes de la EM, y suele estar acompañada de calambres, dolor, disfunción vesical, trastornos del sueño, fatiga y temblor. Recientemente, el concepto de ‘*spasticity-plus syndrome*' (SPS) ha surgido para reflejar la interrelación de estos síntomas. Este trabajo tiene como objetivo actualizar la guía clínica española para el manejo de la espasticidad asociada a la EM (EEM) incluyendo este concepto emergente.

**Métodos:**

Un panel de 14 neurólogos evaluó la evidencia disponible mediante una estrategia de revisión de la literatura a dos niveles, combinando una revisión sistemática sobre el SPS con una *rapid review* abordando el manejo de la EEM. Con base en la guía previa, el panel ha formulado recomendaciones para el manejo de los pacientes con el aval científico de la Sociedad Española de Neurología (SEN). Con el fin de garantizar un proceso de desarrollo estructurado y transparente, se utilizó un marco metodológico adaptado basado en los principios de GRADE para guiar la evaluación de la evidencia y la formulación de las recomendaciones.

**Resultados:**

Un total de nueve publicaciones que abordan el concepto de SPS fueron identificadas. Basadas en la evidencia y el consenso de expertos, el panel desarrolló 13 recomendaciones que cubren los factores desencadenantes y agravantes de la enfermedad, el diagnóstico y evaluación de la espasticidad, y las estrategias terapéuticas para el manejo de los pacientes. Además, se propuso un algoritmo de tratamiento integral para guiar la toma de decisiones terapéuticas en función del perfil clínico de los pacientes.

**Conclusiones:**

Este trabajo proporciona un conjunto actualizado de recomendaciones para el manejo de los pacientes con EEM integrando el concepto de SPS en la práctica clínica. El panel destaca la importancia de llevar a cabo estrategias orientadas a los síntomas que tengan en cuenta el espectro completo de manifestaciones clínicas para individualizar el tratamiento, simplificar el abordaje terapéutico y minimizar el riesgo de polifarmacia.

## Abstract (en)

**Background:**

Multiple sclerosis (MS) is a chronic neuroinflammatory disease that significantly impairs quality of life. Spasticity is one of the most frequent and highly disabling features of MS, often accompanied by cramps, pain, bladder dysfunction, sleep disturbances, fatigue, and tremor. Recently, the concept of ‘spasticity-plus syndrome’ (SPS) has emerged to reflect the interplay among these symptoms. This work aims to update the Spanish clinical guideline for managing MS-related spasticity (MSS) by adopting this evolving concept.

**Methods:**

A panel of 14 Spanish neurologists assessed the available evidence using a two-level literature review strategy, combining a systematic review focused on SPS with a *rapid review* of MSS management. Building on the previous guideline, the panel formulated updated recommendations with the scientific endorsement of the Spanish Society of Neurology (SEN). To support a structured and transparent development process, an adapted GRADE-informed framework was used to guide the appraisal of the evidence and formulation of recommendations.

**Results:**

A total of nine publications addressing the SPS concept were identified. Based on the evidence and expert consensus, the panel developed 13 recommendations covering triggering and aggravating factors, the diagnosis and evaluation of spasticity, and therapeutic strategies for patient management. In addition, a comprehensive treatment algorithm was proposed to guide therapeutic decision-making according to patients’ clinical profiles.

**Conclusions:**

This work provides updated recommendations for the management of patients with MSS, integrating the SPS concept into clinical practice. The panel highlights the importance of a symptom-oriented approach that considers the full spectrum of clinical manifestations to individualize treatment, simplify the therapeutic approach, and minimize the risk of polypharmacy.

## 1. Introducción

La esclerosis múltiple (EM) es una enfermedad neuroinflamatoria, autoinmune y crónica, caracterizada por una desmielinización progresiva del Sistema Nervioso Central (SNC) [1]. En España, su prevalencia se estima en 123,5 casos por cada 100.000 habitantes, reflejando una carga clínica considerable [2]. La esclerosis múltiple recurrente remitente (EMRR) constituye el fenotipo más frecuente de la enfermedad (aproximadamente el 85% de los casos) [3]. El 15% restante de los pacientes presenta esclerosis múltiple primaria progresiva (EMPP), un fenotipo asociado a dificultades tanto diagnósticas como de tratamiento, así como a un pronóstico desfavorable [4]. Aproximadamente el 70% de los pacientes con EMRR progresan eventualmente a una esclerosis múltiple secundaria progresiva (EMSP), que presenta un deterioro gradual e irreversible de las funciones neurológicas [5].

Entre las diversas manifestaciones neurológicas, la espasticidad representa uno de los síntomas más prevalentes e incapacitantes, afectando hasta al 80% de los pacientes en algún momento del transcurso de la enfermedad [6]. Además del incremento en el tono muscular, la espasticidad está acompañada frecuentemente de síntomas como calambres, dolor, disfunción de vejiga, trastornos del sueño, fatiga y temblores [7,8]. Como respuesta a esta complejidad clínica, se ha propuesto un concepto clínico definido como *Spasticity-Plus Syndrome* (SPS) [7]. Esta definición conceptualiza la espasticidad no sólo como una alteración motora aislada, sino como el componente central de un conjunto más amplio e interrelacionado de síntomas [7]. El término SPS ha adquirido una aceptación creciente en la práctica clínica, apoyado por publicaciones que señalan que estos síntomas suelen coexistir y compartirían mecanismos fisiopatológicos subyacentes. Esto sugiere que podrían responder a estrategias terapéuticas comunes [9,10,11].

La guía española más reciente sobre el manejo de la espasticidad en el contexto de la EM data de 2013 [12], por lo que no aborda específicamente el concepto de SPS. Asimismo, la utilización simultánea de varios fármacos para tratar dichos síntomas de forma independiente ha aumentado la preocupación sobre la seguridad, interacciones entre fármacos y adherencia al tratamiento [13,14,15]. Sin embargo, la ausencia de criterios estandarizados para evaluar la espasticidad y sus síntomas asociados, así como los desafíos que presentan los pacientes al describir su sintomatología, han contribuido a una variabilidad significativa tanto en el diagnóstico como en la monitorización clínica [16].

En respuesta a estas carencias, este documento presenta una actualización de las recomendaciones para el abordaje de este síndrome en pacientes con EM. Elaboradas por un panel de neurólogos con experiencia en el manejo de la EM, estas recomendaciones integran la evidencia actual, la experiencia clínica y el nuevo concepto de SPS. El presente documento actualiza el consenso nacional de 2013 sobre el manejo de la espasticidad en pacientes con EM, integrando los avances recientes en fisiopatología, evaluación y tratamiento, e incorporando de forma explícita el concepto emergente de SPS.

A través de la formulación de recomendaciones estructuradas y un algoritmo terapéutico práctico, se busca proporcionar una herramienta clínica que promueva la homogeneización de la práctica asistencial, mejore la toma de decisiones clínicas y contribuya al abordaje integral y personalizado de los pacientes con EM.

## 2. Materiales y Métodos

### 2.1 Diseño del Proyecto

Un panel de catorce renombrados neurólogos participó en el proyecto, de los cuales 7 integraron el comité científico encargado de su coordinación. Los participantes fueron seleccionados debido a una experiencia clínica consolidada en el manejo de pacientes con espasticidad en EM. Se valoraron criterios como una trayectoria asistencial superior a cinco años, participación en proyectos de investigación, publicación de artículos científicos, y la colaboración previa en guías clínicas nacionales o regionales.

El proyecto se estructuró en 4 fases consecutivas: (1) revisión del documento de consenso previo de 2013 [12]; (2) revisión de la literatura estructurada en dos niveles (revisión sistemática de la literatura [RSL] y *rapid review*); (3) evaluación de la evidencia; y (4) formulación y graduación de las recomendaciones.

### 2.2 Revisión del Documento de Consenso de 2013

El proyecto se inició con una reunión virtual en la que el comité científico analizó de forma estructurada el consenso publicado en 2013 [12] valorando la vigencia del contenido y las recomendaciones que precisaban de una revisión. Las conclusiones extraídas de esta primera reunión orientaron la estrategia de búsqueda de la literatura.

### 2.3 Revisión de la Literatura

Con el objetivo de incluir la evidencia clínica más relevante, se realizó una revisión exhaustiva de la literatura comprendida entre 2013–2025 mediante un enfoque en dos etapas. En primer lugar, se llevó a cabo una revisión sistemática de la literatura (RSL) para recopilar específicamente las publicaciones que abordan el concepto de SPS, siguiendo las guías *PRISMA* y *Cochrane* [17,18]. PRISMA checklist provided as **Material Suplementario**. La búsqueda se realizó en las bases de datos internacionales *PubMed*/*Medline* y la librería de *Cochrane* y la base de datos nacional MEDES utilizando una combinación de términos médicos *MeSH* y palabras clave en texto libre (**Tabla Suplementaria 1**). Debido a la novedad y escasa literatura disponible sobre SPS, no se aplicaron restricciones en cuanto al diseño del estudio, el entorno clínico ni la población analizada. Fueron analizados estudios observacionales, ensayos clínicos fase III o IV, revisiones sistemáticas, metaanálisis, guías de práctica clínica y recomendaciones que abordaran el manejo de la espasticidad y sus síntomas asociados en pacientes con EM tanto en inglés como en español.

En segundo lugar, se llevó a cabo una *rapid review* de la literatura para incluir los aspectos transversales del manejo de la espasticidad en EM, incluyendo las herramientas de evaluación, las estrategias de seguimiento y las aproximaciones terapéuticas. Esta metodología se eligió por su utilidad para sintetizar información prioritaria en plazos de tiempo limitados [19] y ha sido validada para el desarrollo de políticas sanitarias públicas y guías clínicas [20]. La revisión no se diseñó para ser tan exhaustiva como una RSL, por lo que no se llevó a cabo un procedimiento de identificación de riesgo de sesgo. La búsqueda se llevó a cabo en la base de datos *Pubmed*/*Medline*, empleando filtros de búsqueda y términos médicos *MeSH *y palabras clave en texto libre combinados con los operadores booleanos “*AND*” y “*OR*” (**Tabla Suplementaria 1**). La selección de estudios se llevó a cabo independientemente por dos revisores, resolviendo las discrepancias por consenso. El cribado se llevó a cabo en dos etapas: una primera revisión de los títulos y resúmenes de las publicaciones, seguida de una evaluación del texto completo.

### 2.4 Evaluación de la Evidencia

La evidencia disponible fue evaluada mediante un marco metodológico estructurado basado en principios clave de la metodología GRADE [21]. No obstante, dada la naturaleza del proyecto, que combinó una revisión sistemática de la literatura, una *rapid review* de la evidencia y un proceso de consenso de expertos, no se llevó a cabo una evaluación GRADE completa, sino adaptada. En su lugar, el panel de expertos valoró de forma global el conjunto de la evidencia que sustentaba cada recomendación y, sobre la base de dicha evaluación, la clasificó como alta, moderada, baja o muy baja. Estas categorías tuvieron como finalidad proporcionar una valoración global de la evidencia más que establecer una calificación formal según la metodología GRADE. Posteriormente, criterios como el riesgo de sesgo en el diseño y ejecución del estudio, inconsistencia de los resultados, evidencia indirecta, imprecisión y potencial sesgo de publicación permitieron ajustar los niveles de calidad asignados [22] (Tabla 1).

**Tabla 1.** **Niveles de calidad de evidencia**.

*Alto*: Alta confianza en la coincidencia entre el efecto real y el estimado.
*Moderado*: Moderada confianza en la estimación del efecto. Hay posibilidad de que el efecto real esté alejado del efecto estimado.
*Bajo*: Confianza limitada en la estimación del efecto. El efecto real puede estar lejos del estimado.
*Muy Bajo*: Poca confianza en el efecto estimado. El efecto verdadero muy probablemente sea diferente del estimado.

### 2.5 Redacción de las Recomendaciones

Tras evaluar la calidad de la evidencia, los miembros del comité científico se reunieron siguiendo la metodología del grupo focal [23] con el objetivo de formular la lista de recomendaciones con base en la evidencia científica y su propia experiencia. La sesión se llevó a cabo de manera online y fue dirigida por un moderador siguiendo un guion semiestructurado. Aunque la calidad de la evidencia disponible para algunas recomendaciones fue catalogada como baja o muy baja, la amplia experiencia clínica de los participantes permitió reforzar el grado de dichas recomendaciones.

### 2.6 Consenso y Grado de Recomendación de las Propuestas Consensuadas Finales

La lista final de recomendaciones se consensuó mediante la metodología de grupos nominales [24]. El panel de 14 participantes fue dividido en 2 grupos nominales, cada uno compuesto por 7 miembros, dentro del rango comúnmente definido (6–12 participantes), para el grupo nominal [25,26]. Todos los participantes recibieron una versión revisada de las recomendaciones y aportaron sus modificaciones al contenido. Basándose en la evidencia y la calidad de esta, evaluaron anónimamente a través de una plataforma online el grado de recomendación de cada propuesta como fuerte o débil/condicional en función del potencial beneficio clínico obtenido [22] (Tabla 2). Se recopiló la información en un documento final donde se aprobaron las recomendaciones y su correspondiente grado de recomendación presentadas en este trabajo. Aquellas recomendaciones que alcanzaron al menos un 66% de consenso por parte de los participantes fueron incluidas en el documento final. El umbral de consenso fue establecido por el comité científico, y se encuentra en línea con los umbrales empleados en documentos de consenso con diseños similares [27].

**Tabla 2.** **Grados de recomendación**.

*Fuerte*: Se espera que la mayoría de los pacientes y profesionales estén de acuerdo con la intervención propuesta. Debe ser implementada en la práctica clínica general y adoptada como política sanitaria en la mayoría de los contextos.
*Débil/Condicional*: Se anticipa una mayor variabilidad en las preferencias de los pacientes. Es necesario un proceso compartido de toma de decisiones y una evaluación individualizada. Las autoridades sanitarias deberán considerar su viabilidad junto con los grupos de interés.

Los datos generados en este artículo se muestran en el mismo y el **Material Suplementario**. El protocolo del estudio se encuentra tras solicitud razonable al autor de correspondencia.

## 3. Resultados

### 3.1 Revisión Sistemática de la Literatura

Se identificaron un total de 9 publicaciones que abordan el concepto de SPS y que fueron incluidas en el análisis final (Fig. 1). Las características de los 9 artículos incluidos (año de publicación, tipo de publicación y tipo de estudio) se encuentran detalladas en la **Tabla Suplementaria 2**.

**Fig. 1.**
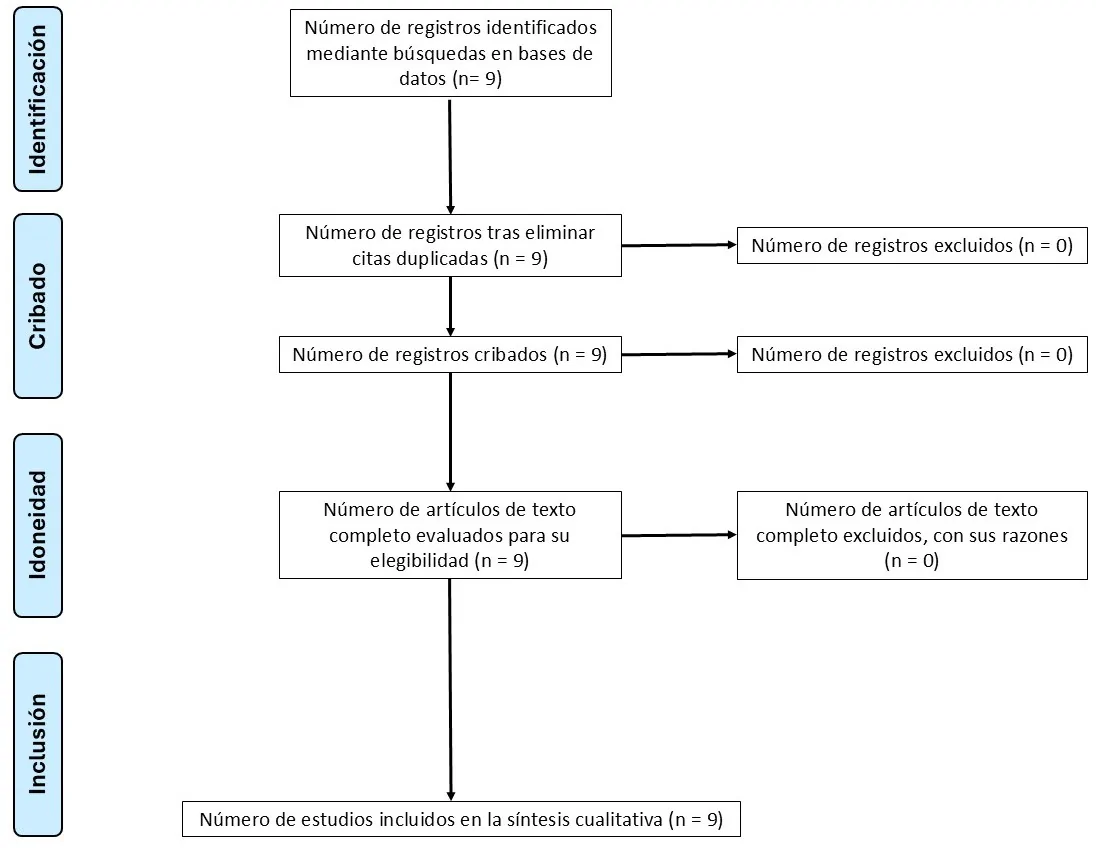
**Diagrama de flujo de PRISMA**. Diagrama de flujo indicando los estudios incluidos.

### 3.2 Recomendaciones de Consenso

Un total de 13 recomendaciones fueron formuladas (Tabla 3), pertenecientes a tres bloques de contenido: (1) una relativa a los factores desencadenantes y agravantes del SPS; (2) seis orientadas al diagnóstico y la evaluación de la espasticidad; y (3) seis centradas en el abordaje terapéutico del SPS.

**Tabla 3.** **Recomendaciones consensuadas**.

Recomendaciones	Calidad de la evidencia	Grado de recomendación
1. Se recomienda considerar tanto los factores inherentes a la EM (incluidos los derivados de su progresión y discapacidad), como los factores externos, al evaluar la espasticidad y sus síntomas asociados.	BAJA	FUERTE
2. Se recomienda ofrecer de manera activa, precisa y adaptada al paciente, información sobre la espasticidad y los síntomas asociados, asegurando una comprensión realista de su impacto y manejo tanto en el momento del diagnóstico como durante el seguimiento.	MODERADA	FUERTE
3. Se recomienda explorar de forma sistemática todos los síntomas asociados a la espasticidad, incluyendo aquellos menos evidentes, para no infraestimar su impacto clínico y mejorar el seguimiento del paciente con EM.	MUY BAJA	FUERTE
4. Se recomienda realizar una evaluación clínica sistemática de la espasticidad y sus síntomas asociados en tres momentos clave: en el momento del diagnóstico, antes de iniciar un nuevo tratamiento, y cada seis meses en pacientes con tratamiento estable y bien controlados. Adicionalmente, se recomienda su evaluación ante cualquier evento clínico relevante que pueda modificar su estado funcional.	MUY BAJA	FUERTE
5. Se recomienda evaluar la espasticidad en los pacientes con EM mediante la Escala de Ashworth Modificada (MAS, de sus siglas en inglés *Modified Ashworth Scale*) y la Escala Numérica de Espasticidad (NRS-S, de sus siglas en inglés *Numerical Rating Scale for Spasticity*).	MODERADA	FUERTE
6. Cuando se considere clínicamente oportuno, se recomienda complementar la evaluación de la espasticidad utilizando la escala de espasmos de Penn (PSFS, de sus siglas en inglés *Penn Spasm Frequency Scale*) y/o la escala de Tardieu modificada (MTS, de sus siglas en inglés *Modified Tardieu Scale*), con el objetivo de obtener una valoración más precisa de fenómenos como los espasmos involuntarios o la hipertonía dependiente de la velocidad.	MODERADA	DÉBIL
7. Se recomienda utilizar la herramienta IDSPS (del inglés *Instrument for the early detection of the Spasticity-Plus Syndrome*) para la identificación del SPS. Asimismo, en casos seleccionados, se recomienda utilizar escalas específicas para cada síntoma predominante con el fin de obtener una valoración más precisa.	BAJA	DÉBIL
8. Se recomienda la elaboración de un plan terapéutico y formativo individualizado para cada paciente.	MUY BAJA	FUERTE
9. En pacientes con EM, los objetivos del manejo terapéutico de la espasticidad y sus síntomas asociados deben centrarse en mejorar la función, especialmente la postura, la marcha y la movilidad, con el objetivo de aliviar síntomas, facilitar las actividades diarias y prevenir complicaciones como úlceras por presión, contracturas o subluxaciones. Es importante considerar que cierto grado de espasticidad puede ser beneficioso al permitir la bipedestación en algunos pacientes.	BAJA	FUERTE
10. La elección del tratamiento farmacológico debe basarse en una evaluación integral que contemple la espasticidad, los síntomas asociados, el historial terapéutico y las características individuales del paciente.	BAJA	FUERTE
11. A la hora de definir el tratamiento más adecuado para el paciente, es necesario evaluar su posible impacto sobre otros síntomas coexistentes que podrían empeorar a de optimizar el balance beneficio-riesgo y evitar la agravación clínica.	BAJA	FUERTE
12. Se recomienda simplificar el tratamiento sintomático minimizando la polifarmacia, el riesgo de efectos adversos y las interacciones farmacológicas siempre que sea posible.	MODERADA	FUERTE
13. Se recomienda evaluar periódicamente la eficacia del tratamiento farmacológico y, en caso de no obtener mejoría significativa, plantear su retirada.	MODERADA	FUERTE

EM, esclerosis múltiple; IDSPS, instrumento para la detección temprana del SPS; MAS, escala de Ashworth modificada; MTS, escala de Tardieu modificada; PSFS, escala de espasmos de Penn; SPS, *spasticity-plus syndrome*.

A continuación, se detallan cada una de las recomendaciones, la evidencia que las sustenta y el grado de recomendación por bloque.

#### 3.2.1 Factores Desencadenantes y Agravantes de la Espasticidad y sus Síntomas Asociados

*Recomendación 1. Se recomienda considerar tanto los factores inherentes a la EM (incluidos los derivados de su progresión y discapacidad), como los factores externos, al evaluar la espasticidad y sus síntomas asociados.*

*Calidad de la evidencia BAJA; Grado de recomendación FUERTE (13/14 participantes)*

A pesar de la falta de estudios sobre los factores inherentes y extrínsecos de la EM, algunos autores han demostrado que la espasticidad no debe entenderse como un fenómeno aislado, sino condicionado por múltiples variables [28,29]. Por este motivo, resulta fundamental realizar una evaluación integral que permita distinguir si el empeoramiento del cuadro clínico se debe a la progresión de la enfermedad o a la influencia de estos factores (Tabla 4). En una encuesta reciente realizada a gran escala en población con EM, aproximadamente la mitad de los pacientes identificaron fatiga, ejercicio físico, cambios de temperatura y estrés como desencadenantes frecuentes de los episodios espásticos [16]. Asimismo, un grupo multidisciplinar ha propuesto un modelo conceptual integrador, en el que dichos factores se caracterizan como precipitantes, y se incorporan además variables moduladoras relacionadas con las características individuales del paciente y con la propia enfermedad, como su duración o el tipo de EM [29]. Este enfoque permite comprender mejor la variabilidad clínica de los síntomas y orientar estrategias terapéuticas más individualizadas y eficaces que favorezcan un mejor control sintomático y una mejor calidad de vida para el paciente. Aunque la evidencia formal es limitada, la experiencia clínica de los expertos y la plausibilidad en la práctica rutinaria justifican la fuerza de la recomendación.

**Tabla 4.** **Factores inherentes a la enfermedad y los factores agravantes de la espasticidad**.

Intensidad	Factores propios de la enfermedad	Complicaciones derivadas de la discapacidad	Factores externos
Alta	• Brotes	• Posturas incorrectas y prolongadas	• Fármacos no específicos de la EM (ej: neurolépticos, antidepresivos, algunos antibióticos…)
	• Progresión de la enfermedad	• Infecciones	• Fármacos específicos de la EM: Interferones
		• Estrés psicológico	• Fiebre
		• Estímulos dolorosos	
		• Trastornos del sueño	
		• Retención urinaria	
Media		• Fatiga excesiva	• Cambios de temperatura
		• Úlceras por presión	• Frío extremo
		• Deshidratación	• Menstruación
Baja		• Uso de sondas vesicales	• Ropa ajustada
		• Fecalomas	• Consumo excesivo de cafeína

EM, esclerosis multiple.

#### 3.2.2 Diagnóstico y Evaluación de la Espasticidad y sus Síntomas Asociados

*Recomendación 2. Se recomienda ofrecer de manera activa, precisa y adaptada al paciente, información sobre la espasticidad y los síntomas asociados, asegurando una comprensión realista de su impacto y manejo tanto en el momento del diagnóstico como durante el seguimiento.*

*Calidad de la evidencia: MODERADA Grado de recomendación: FUERTE (12/14 participantes)*

El modelo de atención centrada en el paciente ha cobrado relevancia en los últimos años como estrategia orientada a fomentar la comunicación bidireccional y la implicación activa del paciente en todas las fases del abordaje terapéutico, con el objetivo de mejorar los resultados clínicos [30]. La comunicación efectiva entre el paciente y el personal sanitario ha sido identificada como un elemento clave para favorecer la autonomía del primero, incrementar su adherencia terapéutica y optimizar tanto el control sintomático inmediato como el pronóstico a largo plazo [31]. No obstante, datos procedentes de encuestas realizadas en población con EM reflejan que la calidad del diálogo entre pacientes y profesionales sigue siendo subóptima. En este sentido, el 65% de los pacientes manifiestan sentirse poco o nada preparados para afrontar un eventual desarrollo de espasticidad. Además, al 52% le habría gustado abordar este tema en fases más tempranas de la enfermedad [16]. Esta falta de comunicación efectiva podría estar relacionada, en parte, con la dificultad que presentan algunos pacientes para describir de forma precisa los síntomas que experimentan [29].

En consonancia con estas observaciones, un reciente consenso nacional en el que participaron 55 neurólogos subrayó la necesidad de establecer una comunicación clínica activa, continuada y rigurosa, con el objetivo de mejorar la identificación precoz del SPS y facilitar así un abordaje más eficaz y personalizado de los mismos [9]. Además, una revisión sistemática de ensayos clínicos aleatorizados ha demostrado que proporcionar una información estructurada a los pacientes con EM se asocia con un aumento significativo de su conocimiento sobre la enfermedad y sobre estrategias de manejo disponibles [32]. Por tanto, se recomienda informar al paciente sobre la espasticidad y síntomas asociados de forma clara, progresiva y adaptada a sus capacidades, así como explicarle la importancia de considerar todos los síntomas dentro de un síndrome (*Spasticity-Plus Syndrome*), y no como síntomas no relacionados. Esta información debe ser proporcionada desde el momento del diagnóstico y mantenida de manera longitudinal durante todo el seguimiento clínico, en línea con recomendaciones previas [31].

*Recomendación 3. Se recomienda explorar de forma sistemática todos los síntomas asociados a la espasticidad, incluyendo aquellos menos evidentes, para no infraestimar su impacto clínico y mejorar el seguimiento del paciente con EM.*

*Calidad de la evidencia: MUY BAJA; Grado de recomendación: FUERTE (12/14 participantes) (Recomendación fuerte a pesar de la muy baja calidad de la evidencia basada en el consenso unánime de expertos y la prevención de deterioro clínico significativo)*

En práctica clínica habitual, durante las visitas de seguimiento, es frecuente que la evaluación se limite a síntomas comunicados espontáneamente por el paciente [9]. Sin embargo, esta aproximación puede conducir a una infraestimación del impacto clínico real, especialmente en el contexto de la espasticidad, ya que algunos de sus síntomas asociados, como los trastornos urinarios, se ocultan con frecuencia por estigma y vergüenza. Además, un estudio donde se incluyeron 1872 pacientes con EM (Francia, Alemania, Italia y Reino Unido), mostró que un 35% de los pacientes desconocía la asociación entre la EM y sus síntomas urinarios [33]. A pesar de la limitada evidencia disponible, el panel considera que una valoración sistemática y estructurada de todos los síntomas potencialmente asociados, incluyendo aquellos de menor frecuencia o expresividad clínica, justifica una recomendación fuerte. En este sentido, se ha descrito que manifestaciones como los trastornos de sueño o las alteraciones del control vesical, aunque menos prevalentes, tienen un efecto negativo significativo sobre la calidad de vida de pacientes con espasticidad [34,35].

*Recomendación 4. Se recomienda realizar una evaluación clínica sistemática de la espasticidad y sus síntomas asociados en tres momentos clave: en el momento del diagnóstico, antes de iniciar un nuevo tratamiento, y cada seis meses en pacientes con tratamiento estable y bien controlados. Adicionalmente, se recomienda su evaluación ante cualquier evento clínico relevante que pueda modificar su estado funcional.*

*Calidad de la evidencia: MUY BAJA; Grado de recomendación: FUERTE (12/14 participantes) (Recomendación fuerte a pesar de la muy baja calidad de la evidencia basada en el consenso unánime de expertos y la prevención de deterioro clínico significativo)*

La guía actual de la Sociedad Española de Neurología (SEN), al igual que otras guías europeas como la *European Academy of Neurology* (EAN) y el *National Institute for Health and Care Excellence* (NICE), no establece intervalos específicos para la evaluación de síntomas como la espasticidad, dejando esta decisión a criterio del profesional responsable. Debido a ello, hay una ausencia de evidencia robusta que especifique cómo y cuándo evaluar la espasticidad. No obstante, respaldado por las recomendaciones de la *American Academy of Physical Medicine and Rehabilitation* (AAPM&R) y la experiencia del panel, se recomienda realizar una valoración estructurada de la espasticidad en el momento del diagnóstico y antes de iniciar cada intervención terapéutica para seleccionar el enfoque más adecuado en cada caso [36].

Por su parte, el *Consortium of Multiple Sclerosis Center*, con el aval de la *American Academy of Neurology*, ya propuso que la espasticidad sea evaluada de manera rutinaria, independientemente de que el paciente la refiera de forma espontánea. Esta guía incluye además un algoritmo clínico en el que se recomienda su valoración en cada visita de seguimiento, especialmente ante la aparición de cambios funcionales [37]. Dado que la espasticidad puede presentar una evolución progresiva o un curso fluctuante, un panel nacional ha considerado que los intervalos habituales de seguimiento resultan excesivamente prolongados para detectar modificaciones clínicas de carácter leve [9]. En este sentido, se propone realizar controles con una frecuencia inferior a seis meses tras el diagnóstico de espasticidad, con el fin de realizar una monitorización más estrecha y una intervención terapéutica oportuna. Este intervalo deriva de la recomendación de expertos para llevar a cabo un manejo balanceado entre monitorización y carga al paciente.

*Recomendación 5. Se recomienda evaluar la espasticidad en los pacientes con EM mediante la Escala de Ashworth Modificada (MAS, de sus siglas en inglés Modified Ashworth Scale) y la Escala Numérica de Espasticidad (NRS-S, de sus siglas en inglés Numerical Rating Scale for Spasticity).*

*Calidad de la evidencia: MODERADA; Grado de recomendación: FUERTE (13/14 participantes)*

Con el objetivo de facilitar la toma de decisiones terapéuticas adecuadas, se recomienda el uso de herramientas estandarizadas que permitan una evaluación sistemática y objetiva de la espasticidad. Entre las guías europeas las herramientas referidas con mayor frecuencia son la MAS y la NRS-S [38]. La MAS es la herramienta más utilizada para cuantificar el aumento en tono muscular debido a su simplicidad y aplicabilidad en la práctica clínica diaria [39]. Además, un metaanálisis confirmó una fiabilidad aceptable en su uso, especialmente en la evaluación de espasticidad en miembros superiores [40]. Asimismo, en un estudio transversal reciente se observó que los valores obtenidos mediante la MAS se correlacionaban de manera significativa con parámetros funcionales como velocidad de la marcha y el equilibrio [8], lo que refuerza su utilidad como herramienta clínica para estimar el impacto funcional de la espasticidad. De forma complementaria, se recomienda el empleo de la escala NRS-S, en línea con las recomendaciones de gran parte de las guías europeas [38], que abogan por la incorporación de medidas centradas en el paciente, validadas y de fácil aplicación, para estandarizar los resultados reportados en la práctica clínica. Además, la escala NRS-S es especialmente útil, ya que se ha demostrado que una reducción ≥30% refleja una diferencia clínicamente importante, mientras que una reducción ≥18% corresponde a la mínima diferencia clínicamente relevante [41].

*Recomendación 6. Cuando se considere clínicamente oportuno, se recomienda complementar la evaluación de la espasticidad utilizando la escala de espasmos de Penn (PSFS, de sus siglas en inglés Penn Spasm Frequency Scale) y/o la escala de Tardieu modificada (MTS, de sus siglas en inglés Modified Tardieu Scale), con el objetivo de obtener una valoración más precisa de fenómenos como los espasmos involuntarios o la hipertonía dependiente de la velocidad.*

*Calidad de la evidencia: MODERADA; Grado de recomendación: DÉBIL (12/14 participantes)*

La espasticidad constituye una manifestación neurológica compleja que no se limita al aumento del tono muscular, sino que engloba también fenómenos como hipertonía dependiente de la velocidad, hiperrespuesta a estímulos externos y aparición de espasmos involuntarios [42]. En este contexto, se recomienda la utilización de herramientas estandarizadas que permitan evaluar de forma específica estos signos clínicos, contribuyendo así a una caracterización más precisa del síndrome espástico. Entre estas herramientas, la escala de Penn ha sido empleada para evaluar la frecuencia de los espasmos [6], y se propone como instrumento complementario en la evaluación clínica rutinaria. Asimismo, esta escala ha sido utilizada para evaluar la eficacia de intervenciones terapéuticas en pacientes con espasticidad asociada a EM [43]. Por otro lado, la MTS, que incorpora la velocidad del estiramiento pasivo en la evaluación del tono muscular, permite discriminar entre espasticidad y retracción muscular, y ha mostrado una buena fiabilidad en la valoración de la espasticidad de los extensores de rodilla en pacientes con EM [44]. Además, su utilidad ha sido demostrada en la evaluación de miembros superiores en adultos con otras afecciones neurológicas [45]. Sin embargo, las dificultades asociadas a la aplicación de estas herramientas en la práctica clínica justifican la debilidad de la recomendación.

*Recomendación 7. Se recomienda utilizar la herramienta IDSPS (del inglés Instrument for the early detection of the Spasticity-Plus Syndrome) para la identificación del SPS. Asimismo, en casos seleccionados, se recomienda utilizar escalas específicas para cada síntoma predominante a fin de obtener una valoración más precisa.*

*Calidad de la evidencia: BAJA; Grado de recomendación: DÉBIL (10/14 participantes)*

Diversos autores han subrayado la importancia de la identificación precoz del síndrome SPS con el objetivo de optimizar el abordaje terapéutico y favorecer una intervención farmacológica oportuna [7,9]. Esta necesidad responde al hecho de que, en práctica clínica habitual, tanto la espasticidad como otros síntomas asociados suelen detectarse en fases avanzadas del curso clínico, lo que puede limitar la eficacia del tratamiento [46]. En este contexto, se ha destacado el valor de disponer de herramientas estructuradas que permitan un cribado sistemático y homogéneo de los síntomas compatibles con SPS [9]. Con este fin, se recomienda el uso de la herramienta IDSPS, diseñada para facilitar la detección de pacientes con alta probabilidad de presentar este síndrome. Esta herramienta ha mostrado una elevada concordancia con el juicio clínico experto (coeficiente de correlación de Pearson = 0,936) y una buena capacidad discriminativa (área bajo la curva = 0,816), así como niveles satisfactorios de sensibilidad (0,71), especificidad (0,78) y precisión global (0,76) [10]. Aunque esta herramienta se ha desarrollado recientemente y precisa de más estudios para su validación, proporciona una estimación probabilística de la presencia del SPS en función de los síntomas reportados por el paciente, clasificando el riesgo como alto (>60%), moderado (30–60%) o bajo (<30%) [10]. Esta clasificación permite orientar la evaluación clínica y apoyar la toma de decisiones diagnósticas. Para aumentar la precisión del proceso diagnóstico, se recomienda complementar esta herramienta con escalas específicas dirigidas a los síntomas predominantes en cada paciente, como escalas para dolor, alteraciones del sueño, alteraciones urinarias, etc. A pesar de los resultados prometedores, es necesario tener en cuenta que la herramienta IDSPS todavía precisa de más estudios de validación que recomienden en alto grado su uso. Por ello, la herramienta debe complementar, que no sustituir, el análisis clínico exhaustivo y ser validada prospectivamente.

#### 3.2.3 Manejo Terapéutico del SPS

*Recomendación 8. Se recomienda la elaboración de un plan terapéutico y un plan formativo individualizado para cada paciente.*

*Calidad de la evidencia: MUY BAJA; Grado de recomendación: FUERTE (14/14 participantes) (Recomendación fuerte a pesar de la muy baja calidad de la evidencia basada en el consenso unánime de expertos y la prevención de deterioro clínico significativo)*

En consonancia con el documento de consenso nacional previamente publicado [12], se recomienda establecer un plan de manejo individualizado adaptado a las características clínicas y necesidades específicas de cada paciente. Para su adecuada elaboración, es fundamental considerar el historial terapéutico previo, preferencias personales, presencia de complicaciones como dolor o contracturas, así como factores contextuales como la situación socioeconómica. También otros síntomas concomitantes que pueden interferir en el manejo clínico, incluyendo alteraciones de la movilidad, fatiga o trastornos del estado de ánimo.

Aunque la evidencia científica es limitada en relación a planes terapéuticos y formativos orientados al paciente, tanto el manual elaborado por SEMERGEN (Sociedad Española de Médicos de Atención Primaria) como la guía terapéutica de la SEN coinciden en destacar la importancia de proporcionar información clara y suficiente al paciente como paso previo para el diseño de un abordaje terapéutico verdaderamente individualizado [47,48]. El objetivo último debe ser un abordaje integral del SPS, orientado a reducir la influencia de factores desencadenantes y agravantes, e incorporar de forma activa estrategias no farmacológicas como el ejercicio terapéutico y la fisioterapia.

*Recomendación 9. En pacientes con EM, los objetivos del manejo terapéutico de la espasticidad y sus síntomas asociados deben centrarse en mejorar la función, especialmente la postura, la marcha y la movilidad, con el objetivo de aliviar síntomas, facilitar las actividades diarias y prevenir complicaciones como úlceras por presión, contracturas o subluxaciones. Es importante considerar que cierto grado de espasticidad puede ser beneficioso al permitir la bipedestación en algunos pacientes.*

*Calidad de la evidencia: BAJA; Grado de recomendación: FUERTE (14/14 participantes)*

En línea con el documento de consenso nacional previamente publicado [12], se recomienda establecer como objetivo terapéutico principal en el abordaje del SPS la mejora de la función, mediante la reducción de la intensidad del tono muscular alterado, para prevenir la aparición de deformidades musculoesqueléticas y limitaciones funcionales secundarias [49]. En un estudio transversal, se observó que los pacientes con niveles más altos de espasticidad presentaban una probabilidad significativamente mayor de discapacidad, así como una peor calidad de vida global, en relación directa con la presencia de déficits físicos y restricciones en la actividad [50]. Entre los aspectos funcionales más afectados, destaca el control postural, comprometido en aproximadamente dos tercios de los pacientes, con repercusión directa sobre la realización de actividades de la vida diaria y la calidad de vida [51].

Aunque los estudios existentes presentan limitaciones en cuanto a su interpretación, el panel considera fundamental evaluar de forma rigurosa el impacto funcional de los tratamientos prestando especial atención a la movilidad. Una revisión sistemática reciente ha subrayado la necesidad de adoptar estrategias terapéuticas integradas que combinen tratamientos farmacológicos y no farmacológicos, orientadas a lograr un equilibrio entre la reducción del tono muscular y la preservación de las capacidades funcionales [52]. Este equilibrio resulta especialmente relevante, ya que, en pacientes con un grado leve o moderado de espasticidad, ésta puede ejercer un papel beneficioso, contribuyendo al control postural y facilitando la bipedestación [53,54].

Por tanto, aunque la evidencia disponible en este ámbito es aún limitada, se recomienda priorizar intervenciones que preserven la movilidad y la funcionalidad del paciente. Los riesgos de no intervenir (deterioro físico, pérdida de autonomía, deterioro de la calidad de vida) justifican una recomendación fuerte basada en principios de buena práctica clínica.

*Recomendación 10. La elección del tratamiento farmacológico debe basarse en una evaluación integral que contemple la espasticidad, los síntomas asociados, el historial terapéutico y las características individuales del paciente.*

*Calidad de la evidencia: BAJA; Grado de recomendación: FUERTE (14/14 participantes)*

Tanto la guía de práctica clínica de SEN como la del NICE coinciden en recomendar un abordaje terapéutico integral en el manejo de pacientes con EM, basado en la combinación de estrategias farmacológicas y no farmacológicas, con el objetivo de ofrecer soluciones individualizadas y adaptadas a las necesidades específicas de cada paciente [48,55].

Aunque son necesarios más estudios que detallen los criterios clave para la elección del tratamiento, se considera fundamental realizar una evaluación global del paciente que permita definir el abordaje terapéutico más adecuado. Esta evaluación debe contemplar no sólo la severidad de la espasticidad, sino también la presencia de síntomas asociados, los tratamientos previamente utilizados y las características individuales del paciente, tales como trastornos de la marcha, fatiga o limitaciones funcionales [16,56]. En función de estos factores, los objetivos terapéuticos deben orientarse hacia la reducción sintomática, la mejora de los niveles de actividad, tanto activa como pasiva, y la optimización de la calidad de vida del paciente [57,58]. En línea con lo propuesto en la literatura reciente [7], se recomienda llevar a cabo una revisión exhaustiva para identificar la presencia de síntomas relacionados con la espasticidad y su grado de severidad, a fin de priorizar opciones terapéuticas eficaces, bien toleradas, y de mayor simplicidad posible. Este abordaje ha sido propuesto previamente, postulando estrategias terapéuticas capaces de actuar sobre múltiples dianas para atajar varios síntomas a la vez [7,59]. Este abordaje holístico, centrado en las necesidades funcionales del paciente, puede contribuir a preservar la autonomía a largo plazo y mejorar de forma sostenida su calidad de vida.

*Recomendación 11. A la hora de definir el tratamiento más adecuado para el paciente, es necesario evaluar su posible impacto sobre otros síntomas coexistentes que podrían empeorar con el fin de optimizar el balance beneficio-riesgo y evitar la agravación clínica.*

*Calidad de la evidencia: BAJA; Grado de recomendación: FUERTE (14/14 participantes)*

La naturaleza polisintomática de la EM ha conducido, en numerosas ocasiones, a la implementación de estrategias terapéuticas centradas en el abordaje aislado de cada síntoma [60,61,62,63]. Este enfoque fragmentado ha dado lugar a situaciones frecuentes de polimedicación en estos pacientes, lo que no siempre se traduce en una mejoría integral del estado clínico ya que puede incrementar el riesgo de efectos adversos y de interacciones farmacológicas [13]. Cabe destacar que algunos de los tratamientos empleados para controlar síntomas específicos pueden, a su vez, inducir nuevos efectos adversos o exacerbar manifestaciones ya presentes [7,62]. Por ejemplo, el uso de antidepresivos puede conllevar disfunción sexual, o tratamientos para reducir la espasticidad o el dolor pueden empeorar la fatiga [64]. Aunque son escasos los estudios que detallan el empeoramiento de los síntomas debido a la polimedicación, se recomienda realizar una evaluación integral del perfil clínico del paciente. Antes de definir la estrategia terapéutica, debería considerarse de forma explícita la presencia, interrelación y severidad de los distintos síntomas para obtener un mayor beneficio. Este enfoque permite seleccionar opciones terapéuticas con una mejor relación beneficio-riesgo, minimizando el riesgo de agravamiento clínico y evitando intervenciones redundantes o contraproducentes. Aunque la calidad de la evidencia es baja, esta recomendación está sustentada por el consenso de expertos y su plausibilidad clínica.

*Recomendación 12. Se recomienda simplificar el tratamiento sintomático minimizando la polifarmacia, el riesgo de efectos adversos y las interacciones farmacológicas siempre que sea posible.*

*Calidad de la evidencia: MODERADA; Grado de recomendación: FUERTE (14/14 participantes)*

En línea con la recomendación anterior, se sugiere simplificar el esquema terapéutico siempre que sea posible, con el objetivo de reducir los efectos adversos derivados de la polifarmacia. Aunque no existe una definición unánime y universal de polifarmacia, los trabajos recientes coinciden en definirlo como el uso de al menos cinco medicamentos diferentes al día, lo que coincide con el umbral considerado en la práctica clínica habitual [65,66]. El manejo sintomático de la EM requiere con frecuencia de la administración simultánea de múltiples fármacos para el tratamiento de la espasticidad, el dolor neuropático, la fatiga, los trastornos del sueño, o los síntomas depresivos. Este enfoque conlleva un incremento del riesgo de interacciones farmacológicas, tanto entre medicamentos prescritos como entre fármacos de dispensación libre, cuya inclusión en la historia clínica puede ser incompleta o inexistente [67]. Una revisión sistemática de la literatura reportó tasas de polifarmacia en pacientes con EM que oscilan entre el 15% y el 59%, asociadas a un mayor riesgo de interacciones farmacológicas potenciales que se correlacionan con un incremento de la discapacidad, deterioro cognitivo, riesgo de hospitalización, mayor número de recaídas y una calidad de vida reducida [13]. Estos datos han sido confirmados en un estudio observacional que incluyó a más de 600 pacientes, en el que se observaron tasas de polifarmacia superiores al 50%, con más del 60% de los pacientes presentando al menos una interacción farmacológica potencial. Este riesgo fue especialmente elevado en aquellos pacientes con comorbilidades cardiovasculares, neurológicas, ortopédicas, o psiquiátricas [67]. El tratamiento de la espasticidad y sus síntomas asociados representa un desafío clínico adicional, dado que la mayoría de opciones terapéuticas disponibles actúan como depresores del sistema nervioso central. Fármacos como baclofeno, tizanidina, cannabinoides y benzodiacepinas comparten propiedades sedantes y pueden producir efectos sinérgicos no deseados, entre ellos somnolencia excesiva, debilidad muscular o inestabilidad postural [68,69,70,71]. Además, todos ellos presentan perfiles de efectos adversos que pueden verse amplificados en situaciones de coadministración.

En este contexto, diversos autores, al igual que los del presente trabajo, destacan la importancia de simplificar los esquemas terapéuticos no sólo para minimizar el riesgo de efectos adversos debido a la polifarmacia, sino para también favorecer la adherencia al tratamiento [9,59]. Por ello, el uso de alternativas terapéuticas capaces de abordar el SPS en su conjunto se presenta como una opción potencialmente eficaz para optimizar el manejo clínico y mejorar la calidad de vida de los pacientes [59].

*Recomendación 13. Se recomienda evaluar periódicamente la eficacia del tratamiento farmacológico y, en caso de no obtener mejoría significativa, plantear su retirada.*

*Calidad de la evidencia: MODERADA; Grado de recomendación: FUERTE (14/14 participantes)*

En línea con las recomendaciones emitidas por la SEN, se aconseja realizar revisiones periódicas de la eficacia y el balance beneficio-riesgo de los tratamientos farmacológicos prescritos para la espasticidad. Esta práctica permite asegurar la adecuación del tratamiento sintomático a los objetivos terapéuticos individuales y prevenir la cronificación innecesaria de fármacos que no hayan demostrado un beneficio clínico tangible [72]. Esta recomendación se alinea con lo establecido en la guía NICE más reciente sobre el manejo de la EM en adultos, la cual indica que, una vez alcanzada la dosis óptima, debe llevarse a cabo una evaluación anual de la eficacia y la seguridad el tratamiento, y en caso de no observarse beneficios claros, suspender su administración [55]. Más allá de constituir una buena práctica clínica, esta estrategia contribuye a la optimización del abordaje integral del paciente con SPS, al evitar la exposición prolongada a tratamientos ineficaces, reducir la carga farmacológica y facilitar la adaptación dinámica del enfoque terapéutico a las necesidades del paciente. La retirada del tratamiento debe realizarse de forma individualizada y bajo monitorización estrecha, idealmente en coordinación con los equipos de neurología y rehabilitación, a fin de garantizar la seguridad clínica y preservar la funcionalidad del paciente.

### 3.3 Algoritmo Terapéutico

El abordaje terapéutico constituye un eje fundamental en las principales guías clínicas sobre el manejo de la espasticidad en pacientes con EM [36,38,48,55]. Dichas guías recomiendan una intervención individualizada y adaptada a las características específicas de cada paciente, pero en muchos casos no proporcionan un algoritmo terapéutico claramente definido en función del perfil clínico [38]. Ante esta limitación, el comité propone a continuación un algoritmo (**Fig. Suplementaria 1**) orientado a facilitar la toma de decisiones en la práctica clínica rutinaria, contribuyendo a un manejo más estructurado, coherente, y eficiente del SPS en pacientes con EM (Fig. 2; Fig. 3, Ref. [71]; Fig. 4).

**Fig. 2.**
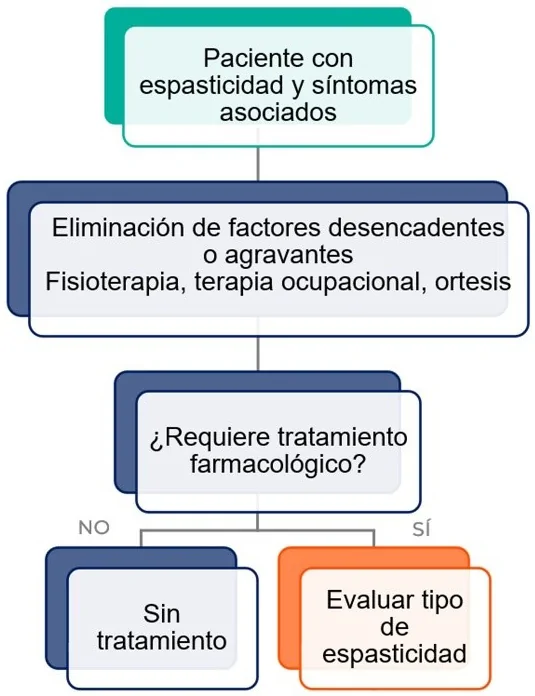
**Algoritmo Terapéutico (inicio)**. Algoritmo desarrollado a partir del consenso de expertos para el tratamiento de pacientes con espasticidad y síntomas asociados en función del perfil clínico. Su uso debe validarse prospectivamente, adaptando el algoritmo al contexto clínico y bajo juicio clínico experto.

**Fig. 3.**
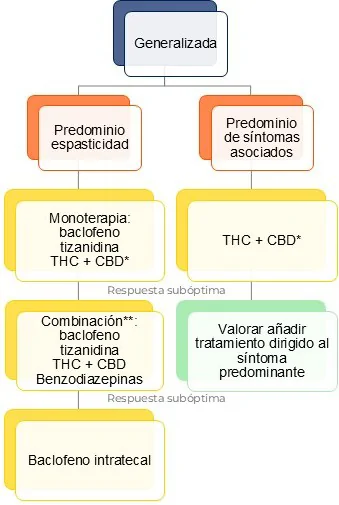
**Algoritmo Terapéutico para espasticidad generalizada**. Algoritmo desarrollado a partir del consenso de expertos para el tratamiento de pacientes con espasticidad y síntomas asociados en función del perfil clínico. Su uso debe validarse prospectivamente, adaptando el algoritmo al contexto clínico y bajo juicio clínico experto. CBD, cannabidiol; THC, tetrahidrocannabinol. * La combinación THC+CBD aprobada en España para los síntomas de EEM está indicada tras respuesta inadecuada a otros medicamentos antiespásticos [71]. En caso de no disponibilidad valorar otra opción. ** Se pueden contemplar distintas combinaciones, no es necesario administrar todos los fármacos de manera conjunta.

**Fig. 4.**
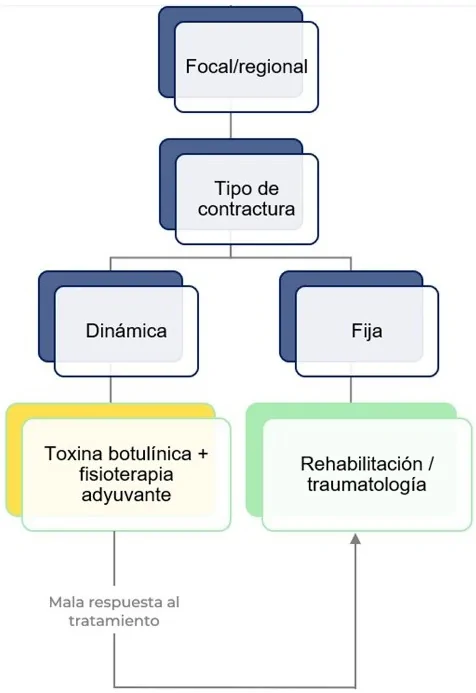
**Algoritmo Terapéutico para espasticidad focal/regional**. Algoritmo desarrollado a partir del consenso de expertos para el tratamiento de pacientes con espasticidad y síntomas asociados en función del perfil clínico. Su uso debe validarse prospectivamente, adaptando el algoritmo al contexto clínico y bajo juicio clínico experto.

Antes de iniciar tratamiento farmacológico, resulta esencial identificar y en su caso, corregir factores desencadenantes o agravantes que puedan contribuir a la exacerbación del cuadro clínico, tales como infecciones, dolor mal controlado, alteraciones posturales, o la suspensión reciente de tratamientos previos, coincidiendo con lo sugerido previamente por otros autores [58,73,74]. El abordaje de estos factores puede conducir a una reducción significativa de la espasticidad sin necesidad de recurrir a tratamiento farmacológico [58,73].

Por ello, se recomienda iniciar tempranamente intervenciones no farmacológicas, como fisioterapia especializada, terapia ocupacional o uso de órtesis funcionales, siempre adaptadas a las necesidades y objetivos individuales del paciente (Fig. 2). Estas estrategias han demostrado su utilidad en el mantenimiento funcional de los pacientes, y presentan un perfil de seguridad favorable, sin riesgo de inducir o agravar otros síntomas [75]. Asimismo, aunque los programas de ejercicio físico pueden contribuir a mitigar síntomas asociados, como la fatiga, la evidencia sobre su efecto directo en la reducción de la espasticidad es todavía limitada y requiere de una mayor validación científica [76,77].

Cuando las medidas previamente descritas resulten insuficientes o la espasticidad evolucione hacia formas clínicas de mayor severidad, debe considerarse el inicio del tratamiento farmacológico. La elección del tratamiento deberá basarse en una evaluación detallada del tipo de espasticidad presente, diferenciando entre formas generalizadas y focales/regionales, con el fin de seleccionar la opción terapéutica más adecuada para cada perfil clínico. A continuación, se presentan las opciones terapéuticas disponibles.

#### 3.3.1 Espasticidad Generalizada

En espasticidad generalizada, debe determinarse si el aumento del tono muscular constituye el síntoma predominante o si éste se presenta en el contexto de un síndrome más complejo con síntomas asociados relevantes (Fig. 3). Cuando la espasticidad representa el componente clínico principal, la opción recomendada sería la monoterapia oral con agentes antiespásticos como baclofeno, tizanidina, o la combinación de tetrahidrocannabinol (THC) y cannabidiol (CBD).

**Baclofeno Oral**: Está indicado para el tratamiento de la espasticidad asociada a EM, lesiones medulares, y otras enfermedades cerebrales de etiología vascular, neoplásicas, degenerativas o desconocida [68]. Una revisión sistemática de ensayos clínicos controlados y estudios observacionales mostró que, en 6 de los 7 estudios analizados, el baclofeno produjo una mejoría significativa del tono muscular, el clonus y los espasmos, con reducciones superiores al 50% en las escalas empleadas para la evaluación de la espasticidad [60]. Su uso puede verse limitado por la aparición de efectos adversos, entre los que destacan somnolencia, debilidad muscular, parestesia, y xerostomía, con tasas de incidencia que oscilan entre el 25% y el 75% de los pacientes tratados [78].

**Tizanidina**: Está indicada en adultos para el tratamiento de la espasticidad secundaria a diversas patologías neurológicas, incluyendo la EM, la mielopatía crónica, los trastornos degenerativos de la médula espinal, los accidentes cerebrovasculares, y la parálisis cerebral [69]. La revisión de los estudios disponibles ha evidenciado que este fármaco ha demostrado producir una mejoría significativa del tono muscular incluso tras la administración de dosis únicas. A medio plazo, se han observado reducciones modestas, aunque no siempre estadísticamente significativas, en la frecuencia de espasmos y clonus [60]. Un metaanálisis reciente que evaluó el uso de antiespásticos orales en enfermedades neurológicas concluyó que la tizanidina es más eficaz que el placebo, mostrando una eficacia comparable a la del baclofeno en el tratamiento de la espasticidad [79]. Su perfil de seguridad está condicionado por su actividad adrenérgica central, lo que se traduce en efectos adversos dosis-dependientes como somnolencia, xerostomía, e hipotensión. También se han descrito casos de sedación significativa y elevación transitoria de enzimas hepáticas, por lo que se recomienda realizar un seguimiento clínico periódico durante su administración [60,80].

**Combinación de THC+CBD**: Está indicada como tratamiento para la mejoría de los síntomas en pacientes adultos con espasticidad moderada o grave debida a la EM que no han respondido de forma adecuada a otros medicamentos antiespásticos y que han mostrado una mejoría clínicamente significativa de los síntomas relacionados con la espasticidad tras una fase inicial de prueba con respuesta clínica significativa [15,71]. Diversas revisiones sistemáticas han respaldado la eficacia de esta combinación en el control de la espasticidad [81,82,83]. En ensayos clínicos aleatorizados, la administración de THC+CBD ha demostrado una reducción significativa en la puntuación de espasticidad medida mediante la escala NRS-S en comparación con el placebo (diferencia media: –0,84 *p* = 0,0002) [80,84]. Estos resultados han sido confirmados en estudios observacionales en condiciones de práctica clínica real, donde se ha observado una reducción del número medio de espasmos diarios del 68,2%, así como mejoras relevantes en síntomas asociados como fatiga (–26,4%) y dolor (–40,4%). También se ha reportado una reducción de los episodios semanales de incontinencia urinaria (–69,3%) en comparación con el valor basal [85,86].

En conjunto, los datos disponibles muestran que alrededor del 40% de los pacientes que responden inicialmente mantienen beneficios clínicamente relevantes durante el seguimiento [87]. Si bien su uso según ficha técnica está indicado en pacientes con respuesta inadecuada a otros antiespásticos, la Sociedad Alemana de Neurología ha otorgado a esta formulación un nivel de recomendación equiparable al de las terapias de primera línea, basándose en la calidad de la evidencia y la experiencia acumulada en el desarrollo clínico [87,88]. Además, un estudio reciente ha concluido que no existen diferencias en la tasa de respuesta al tratamiento en monoterapia o como tratamiento complementario, siendo ambas opciones efectivas, seguras y bien toleradas [89].

En caso de que la respuesta clínica obtenida con monoterapia sea insuficiente, puede considerarse la utilización de esquemas terapéuticos combinados. La asociación de baclofeno, tizanidina, THC+CBD y, en determinados casos, benzodiacepinas, debe valorarse individualmente en función del perfil clínico del paciente, el balance beneficio-riesgo, y la tolerancia a los tratamientos previos. En pacientes que presentan espasmos dolorosos como síntoma predominante, puede contemplarse el uso de fármacos moduladores del dolor neuropático, como la gabapentina, o la pregabalina, por su efecto dual sobre el dolor y los espasmos musculares.

**Gabapentina**: Se ha considerado una opción terapéutica útil en pacientes con EM que presentan espasticidad acompañada de dolor neuropático. En esta población, su administración ha demostrado una reducción significativa tanto de la espasticidad subjetiva como del dolor neuropático [90]. Resultados similares se ha reportado en pacientes con lesión medular, si bien el efecto más destacado se ha observado sobre el componente doloroso y la mejoría en espasticidad es subjetiva [91]. Aunque la gabapentina no se considera un agente antiespástico de primera línea, su uso puede estar indicado en aquellos casos en los que el dolor neuropático constituye un síntoma predominante, especialmente en el contexto de espasmos dolorosos o exacerbaciones agudas.

**Pregabalina**: El uso de pregabalina en el tratamiento de la espasticidad asociada a EM no cuenta actualmente con el respaldo de ensayos clínicos controlados, y la evidencia disponible proviene fundamentalmente de estudios observacionales y de práctica clínica habitual. En una serie retrospectiva de casos que incluyó a 22 pacientes, se observó que 12 de ellos experimentaron mejoría en tono muscular y frecuencia de espasmos, mientras que en 6 pacientes se reportó una reducción específica en el número de espasmos diarios [92]. Estos hallazgos fueron respaldados por un estudio retrospectivo posterior, en el que la reducción de la dosis de pregabalina se asoció con un aumento significativo en las puntuaciones subjetivas de espasticidad (cambio en la mediana de 4 a 6; *p* < 0,01) lo que sugiere un posible efecto beneficioso del tratamiento sobre este síntoma [93]. Si bien la pregabalina no se considera un antiespástico de primera línea, puede valorarse como opción terapéutica en situaciones clínicas específicas, especialmente en el contexto de la espasticidad dolorosa refractaria. No obstante, su uso debe recomendarse con precaución debido a la limitada robustez de la evidencia disponible.

En pacientes con respuesta clínica insuficiente o intolerancia al tratamiento antiespástico por vía oral, puede considerarse el uso de baclofeno intratecal, la cual requiere de bomba implantada y monitorización estrecha. Esta modalidad terapéutica está indicada en adultos con espasticidad crónica grave de origen espinal, como en el contexto de la lesión medular, EM u otras enfermedades de origen cerebral, que no responden de forma adecuada a antiespásticos orales, incluido el baclofeno, o que presentan efectos adversos inaceptables con dosis eficaces por esta vía [8]. La indicación también se extiende a pacientes pediátricos mayores de 4 años y menores de 18 años que presenten estas mismas condiciones clínicas [94]. En estudios clínicos, el baclofeno intratecal ha demostrado una reducción media del 58% en la puntuación de espasticidad medida mediante la escala MAS [95]. Asimismo, un estudio comparativo transversal evidenció que los pacientes tratados con baclofeno intratecal presentaban una frecuencia significativamente menor de espasmos (media de 1,44 frente a 2,37; *p* < 0,01), así como una menor gravedad de estos en comparación a la administración de baclofeno vía oral (media de 1,44 frente a 2,16; *p* < 0,01) medida con la escala PSFS [96].

En pacientes con espasticidad generalizadas acompañada de otros síntomas (SPS) como dolor (incluyendo dolor neuropático y músculo-esquelético asociado a la espasticidad), espasmos, temblor, disfunción vesical, fatiga o alteraciones del sueño, se recomienda considerar el tratamiento con la formulación combinada de THC y CBD. Su perfil terapéutico está respaldado por numerosas revisiones sistemáticas y estudios observacionales en condiciones de práctica real [81,82,83,86]. Los datos disponibles muestran tasas de respuesta clínica que oscilan entre el 40% y el 80% tras cuatro semanas de tratamiento, con una reducción en la NRS-S media mantenida a los 6–12 meses. Un estudio observacional reciente evidenció una reducción media del 40% en la intensidad del dolor, una reducción a 3,4 espasmos/día (–68,2%), y una reducción del 47,2% de los trastornos del sueño en los pacientes tratados [85]. En cuanto a los síntomas urinarios se ha documentado una reducción del 35% en su severidad (*International Prostatic Symptoms Score*). Los datos urodinámicos mostraron los beneficios de la formulación THC+CBD especialmente en los pacientes con vejiga hiperactiva, con una reducción del 50% en la presión del músculo detrusor [97]. Aunque la evidencia sobre su efecto en la fatiga es aún limitada, un estudio reportó que el 61,6% de los pacientes tratados con la formulación THC+CBD alcanzaron el objetivo de reducir la fatiga “mejor de lo esperado” [98].

#### 3.3.2 Espasticidad Focal o Regional

En los casos de espasticidad focal o regional, se recomienda una evaluación inicial del tipo de contractura (Fig. 4). En presencia de una contractura dinámica, podría ser adecuada una primera línea de tratamiento con toxina botulínica tipo A en combinación con un programa de fisioterapia individualizada. La toxina botulínica tipo A está indicada para el tratamiento de la espasticidad focal, así como para la incontinencia urinaria en adultos con hiperactividad neurogénica del detrusor [99,100,101]. Siguiendo las recomendaciones detalladas en el documento de consenso previo [12], se recomienda una dosis máxima en adultos de 1.500 U de Dysport ® (400 U de Botox ®) por sesión de tratamiento y de 250 U de Dysport (50 U de Botox) por punto de inyección. La dosis máxima es de 6 U/kg en músculos grandes y de 2 U/kg en músculos pequeños, sin superar las 300–400 U totales de Dysport (80–100 de Botox) por músculo. Se recomienda inyectar la toxina en mitad del vientre muscular, e infiltrar en varios puntos en músculos grandes y en un único punto en músculos pequeños. Su eficacia en contracturas dinámicas ha sido demostrada tanto en estudios controlados como observacionales, con mejoras sostenidas en el tono muscular, la movilidad y el dolor asociado a la espasticidad [60,102,103,104,105,106]. En un estudio multicéntrico, la administración repetida durante 4 ciclos produjo una mejoría en la prueba de marcha de seis minutos (incremento medio del 11,3% en la distancia recorrida) y una reducción notable de la fatiga [107]. Asimismo, en el estudio GASEPTOX, 205 objetivos de un total de 239 objetivos funcionales definidos (85,8%) fueron conseguidos con el tratamiento [108]. En cuanto a su uso en disfunción vesical, los ensayos clínicos de fase III demostraron reducciones significativas de los episodios de incontinencia semanales, y mejoras en la calidad de vida y los parámetros urodinámicos [109]. En aquellos casos en los que el tratamiento con toxina botulínica resulte insuficiente, o cuando se identifique una contractura crónica desde el inicio, se recomienda derivación temprana a los servicios de rehabilitación o traumatología. Esta derivación permitirá valorar intervenciones quirúrgicas correctivas o enfoques ortopédicos complementarios, con el objetivo de mejorar la funcionalidad y prevenir complicaciones a largo plazo. Además, dicha derivación temprana a rehabilitación o traumatología puede evitar la cronificación.

## 4. Conclusiones

Este documento, fruto del consenso de expertos en EM, formula 13 recomendaciones y un algoritmo terapéutico que cuentan con el aval científico de la SEN, y actualizan y amplían el consenso nacional previo. Un punto diferencial con el consenso previo es la introducción del concepto de SPS, incluyendo la necesidad de considerar todos los síntomas relacionados y susceptibles en muchas ocasiones a un solo fármaco. Se mantiene la recomendación de evaluar factores desencadenantes y agravantes de la espasticidad, ampliando su alcance a los síntomas asociados según el nuevo concepto de SPS [7,29]. Asimismo, se refuerza la necesidad de seguimiento estrecho y se incorpora como novedad la educación al paciente, promoviendo un manejo más personalizado [9,31].

Se recomienda reevaluar la espasticidad al menos cada 6 meses, además de en el diagnóstico y antes de las decisiones terapéuticas. Para la evaluación, se mantienen las escalas MAS y NRS-S, incorporando PSFS y MTS en casos clínicamente relevantes, así como la IDSPS para la identificación del SPS [10]. Se sugiere complementar con escalas específicas según los síntomas.

El consenso introduce también la valoración de los síntomas asociados como criterio clave en la elección del tratamiento [7], subrayando el riesgo de polifarmacia y recomendando reducir fármacos solapados o ineficaces [13,67].

En cuanto al algoritmo terapéutico, se mantiene la evaluación inicial del tipo de espasticidad, pero se añade uno específico para espasticidad generalizada. Si predomina la espasticidad, se propone baclofeno o tizanidina como primera línea, considerando el uso de THC+CBD debido a la evidencia favorable disponible [81,86,88]. Ante respuesta subóptima, se contempla el uso de diversas combinaciones, benzodiacepinas o baclofeno intratecal. En casos con predominancia de los síntomas asociados, se recomienda el uso de THC+CBD, pudiendo complementarse con otros fármacos dirigidos a síntomas concretos.

En espasticidad focal, se actualizan las recomendaciones para el manejo de contracturas dinámicas, sugiriendo la derivación temprana a rehabilitación o traumatología cuando la respuesta a toxina botulínica o fisioterapia adyuvante es insuficiente. Las recomendaciones específicas sobre ejercicio físico y programas de fisioterapia quedan fuera del ámbito de este consenso, por lo que se aconseja valoración por profesionales especializados. Estrategias emergentes como la realidad virtual o los trajes robóticos podrían aportar beneficios adicionales [110,111], aunque se requiere de mayor evidencia para confirmar su eficacia.

Este documento actualiza las recomendaciones nacionales para el manejo de la espasticidad en EM. Se proponen 13 recomendaciones y un algoritmo terapéutico, con énfasis en la identificación de factores desencadenantes, la educación del paciente, la evaluación periódica con herramientas estandarizadas y la detección precoz del SPS. El tratamiento debe individualizarse según el perfil clínico, priorizando la simplificación terapéutica y la prevención de polifarmacia. El consenso destaca la necesidad de integrar opciones farmacológicas y no farmacológicas, adaptadas al síntoma predominante y a la funcionalidad del paciente. Sin embargo, este documento presenta una serie de limitaciones metodológicas que deben ser consideradas. En primer lugar, el desarrollo del algoritmo se ha basado en un consenso de expertos dentro del ámbito español, lo que puede limitar la extrapolación de las conclusiones en otros contextos y sistemas de salud. Esta limitación aplica a tratamientos cuya aprobación y/o indicación puede ser diferente en otros contextos sanitarios. En segundo lugar, aunque se ha integrado evidencia disponible en la literatura nacional e internacional, parte de las decisiones terapéuticas reflejan juicios clínicos basados en la experiencia, debido a la falta de ensayos y guías sobre el SPS. Esto introduce un componente de subjetividad que es inevitable en los documentos de consenso, especialmente en áreas donde la evidencia aún es limitada o emergente, como es el caso. Además, la ausencia de un proceso formal de validación como el método Delphi o la revisión externa independiente podrían considerarse factores que limitan la robustez metodológica del consenso alcanzado. Por último, la evaluación de la evidencia y la formulación de las recomendaciones se basaron en principios de la metodología GRADE sin adherirse estricta y completamente a esta metodología, lo que supone una limitación metodológica.

Pese a las limitaciones mencionadas, el trabajo presenta fortalezas metodológicas relevantes, y constituye una guía práctica y aplicable en la atención de personas con EM y espasticidad. Se ha priorizado la aplicabilidad clínica mediante una evaluación progresiva y adaptable de la respuesta terapéutica, estableciendo criterios para avanzar o modificar la estrategia en función de la evolución clínica. Estas características favorecen la coherencia interna del modelo y su utilidad como herramienta clínica orientativa.

## Contribuciones de los Autores

COG: conceptualización, metodología, medios, escritura del manuscrito original, revisión y edición del manuscrito. JMGD: conceptualización, metodología, medios, escritura del manuscrito original, revisión y edición del manuscrito. PME: conceptualización, metodología, medios, escritura del manuscrito original, revisión y edición del manuscrito. LCF: conceptualización, metodología, medios, escritura del manuscrito original, revisión y edición del manuscrito. MLMG: conceptualización, metodología, medios, escritura del manuscrito original, revisión y edición del manuscrito. MÁLG: conceptualización, metodología, medios, escritura del manuscrito original, revisión y edición del manuscrito. JMPG: conceptualización, metodología, medios, escritura del manuscrito original, revisión y edición del manuscrito. CRT: conceptualización, metodología, medios, escritura del manuscrito original, revisión y edición del manuscrito. AMAT: conceptualización, metodología, medios, escritura del manuscrito original, revisión y edición del manuscrito. CC: conceptualización, metodología, medios, escritura del manuscrito original, revisión y edición del manuscrito. FPM: conceptualización, metodología, medios, escritura del manuscrito original, revisión y edición del manuscrito. SE: conceptualización, metodología, medios, escritura del manuscrito original, revisión y edición del manuscrito. TCT: conceptualización, metodología, medios, escritura del manuscrito original, revisión y edición del manuscrito. ABC: conceptualización, metodología, medios, escritura del manuscrito original, revisión y edición del manuscrito. Todos los autores aprueban la versión final del mismo. Todos los autores leyeron y aprobaron la versión final del manuscrito. Todos los autores participaron de manera suficiente en el trabajo y aceptan ser responsables de todos los aspectos del mismo.

## Aprobación Ética y Consentimiento Informado

No aplica.

## Material Suplementario

El material suplementario asociado con este artículo se puede encontrar, en la versión en línea, en https://doi.org/10.31083/RN51172.
