# Supplementary material for: Spasticity in Multiple Sclerosis: 2026 Expert Consensus
Source: Rev Neurol. 2026 Oct 27;81(10):51172. [Article in Spanish] doi: 10.31083/RN51172 (PMC13732422; doi:10.31083/RN51172)
Supplement: Supplementary file 1 [file 1576-6578-81-10-51172-s1.zip › Supplementary Material.docx]

Material Suplementario

**Tabla Suplementaria 1.** Términos y estrategia de búsqueda

| **Rapid Review** | |
| --- | --- |
|  | **Bases de datos consultadas** |
|  | PubMed/MEDLINE |
|  | **Periodo de búsqueda** |
|  | Enero 2013 - Enero-2025 |
|  | **Idiomas** |
|  | Ingles y Español |
|  | **Población** |
|  | Adultos con EM |
|  | **Estudios elegibles** |
|  | Guías de práctica clínica |
|  | Revisiones sistemáticas |
|  | Metaanálisis |
|  | Ensayos clínicos aleatorizados |
|  | Estudios observacionales |
|  | Documentos de consenso |
|  | **Criterios de inclusión** |
|  | Pacientes adultos con EM |
|  | Estudios que evalúan la espasticidad o la espasticidad y sus síntomas asociados |
|  | Estudios sobre diagnóstico, evaluación, seguimiento o tratamiento |
|  | **Criterios de exclusión** |
|  | Poblaciones exclusivamente pediátricas |
|  | Estudios con animales o preclínicos |
|  | Series de casos |
|  | Resúmenes de conferencias sin artículos completos asociados |
|  | Estudios que no abordan espasticidad en EM |
|  | Publicaciones sin información relevante sobre el manejo de la espasticidad |
|  | **Términos relacionados con espasticidad** |
| #1 | “Spasticity-related"[Title/Abstract] |
| #2 | "Muscle Spasticity"[MeSH] |
| #3 | "Muscle Spasticity"[Title/Abstract] |
| #4 | "Multiple sclerosis-related spasticity" |
|  | **Términos relacionados con EM** |
| #5 | “Multiple sclerosis"[Title/Abstract] |
| #6 | "Multiple sclerosis"[Mesh] |
|  | **Términos relacionados con los factores de origen de la espasticidad** |
| #7 | “Origin factors"[Title/Abstract] |
| #8 | "Activation factors"[Title/Abstract] |
| #9 | “Etiology" [Subheading] |
| #10 | "Etiology"[Title/Abstract]) |
|  | **Términos relacionados con la sintomatología de la espasticidad** |
| #11 | "Symptoms"[Title/Abstract] |
| #12 | "Signs and Symptoms"[Mesh] |
| #13 | "Signs and Symptoms"[Title/Abstract] |
|  | **Términos relacionados con el diagnóstico de la espasticidad** |
| #14 | "Diagnosis"[Mesh] |
| #15 | "Diagnos*"[Title/Abstract] |
| #16 | "Diagnoses and examinations"[Title/Abstract] |
|  | **Términos relacionados con la evaluación de la espasticidad** |
| #17 | “Spasticity assessment"[Title/Abstract] |
| #18 | “Spasticity scales”[Title/Abstract] |
| #19 | “Spasticity measure”[Title/Abstract]) |
|  | **Términos relacionados con la calidad de vida de la espasticidad** |
| #20 | "Quality of Life"[Mesh] |
| #21 | "Quality of Life"[Title/Abstract] |
| #22 | “Health-Related Quality Of Life"[Title/Abstract] |
| #23 | “HRQOL”[Title/Abstract] |
|  | **Términos relacionados con la terapia de la espasticidad** |
| #24 | “Therap*"[Title/Abstract] |
| #25 | “Drug therapy"[Title/Abstract] |
| #26 | “Treatment"[Title/Abstract] |
| #27 | “Spasticity management”[Title/Abstract] |
| #28 | “Management of multiple sclerosis”[Title/Abstract] |
| #29 | "Physical Therapy Modalities"[Mesh] |
| #30 | "Physical Therapy Modalities"[Title/Abstract] |
| #31 | “Physical Therapy Technique"[Title/Abstract] |
| #32 | “Rehabilitation"[Title/Abstract] |
|  | **Estrategia de búsqueda** |
|  | ((1 OR 2 OR 3 OR 4) AND (5 OR 6)) AND ((7 OR 8 OR 9 OR 10) OR (11 OR 12 OR 13) OR (14 OR 15 OR 16) OR (17 OR 18 OR 19) OR (20 OR 21 OR 22 OR 23) OR (24 OR 25 OR 26 OR 27 OR 28 OR 29 OR 30 OR 31 OR 32)) |
|  |  |
| **RSL** |  |
|  | **Términos relacionados con el SPS** |
| #1 | “Spasticity-plus syndrome” |
|  | **Términos relacionados con la EM** |
| #2 | “multiple sclerosis"[Title/Abstract] |
| #3 | "multiple sclerosis"[Mesh] |
| #4 | "multiple sclerosis-related spasticity"[Title/Abstract] |
|  | **Estrategia de búsqueda** |
|  | 1 AND (2 OR 3 OR 4) |
|  | "Spasticity-plus syndrome" AND ("multiple sclerosis"[Title/Abstract] OR "multiple sclerosis"[Mesh] OR "multiple sclerosis-related spasticity"[Title/Abstract]) |

**Tabla Suplementaria 2** Artículos incluidos

| **Autor Principal** | **Año** | **Tipo de publicación** | **Tipo de estudio** | **Referencia** |
| --- | --- | --- | --- | --- |
| **Bruno A (I)** | 2025 | Artículo de revisión | Revisión de la literatura | [1] |
| **Fernández Fernández Ó.** | 2024 | Artículo original | Encuesta | [2] |
| **Goicochea Briceño H** | 2024 | Artículo original | Estudio observacional retrospectivo en pacientes adultos con EM | [3] |
| **Alami Marrouni K** | 2022 | Artículo de opinión | Artículo de opinión | [4] |
| **Patti F** | 2022 | Artículo original | Estudio observacional, multicéntrico, retrospectivo. | [5] |
| **Chan A** | 2022 | Artículo de revisión | Artículo de revisión de ensayos clínicos, estudios de extensión, estudios postaprobación y estudios observacionales | [6] |
| **Bruno A (II)** | 2022 | Artículo de revisión | Artículo de revisión | [7] |
| **Fernández O et al (I)** | 2021 | Artículo original | Exposición de resultados cualitativos y cuantitativos tras una metodología de discusión estructurada (*Workmat R*) | [8] |
| **Fernández O et al (II)** | 2020 | Artículo de revisión | Artículo de revisión | [9] |
| *EM, esclerosis múltiple* | | | | |

**Figura Suplementaria 1 Algoritmo Terapéutico**. Algoritmo desarrollado a partir del consenso de expertos para el tratamiento de pacientes con espasticidad y síntomas asociados en función del perfil clínico. Su uso debe validarse prospectivamente, adaptando el algoritmo al contexto clínico y bajo juicio clínico experto. CBD, cannabidiol; THC, tetrahidrocannabinol. * La combinación THC+CBD aprobada en España para los síntomas de EEM está indicada tras respuesta inadecuada a otros medicamentos antiespásticos [10]. En caso de no disponibilidad valorar otra opción. ** Se pueden contemplar distintas combinaciones, no es necesario administrar todos los fármacos de manera conjunta.

**Referencias**

[1] Bruno A, Annovazzi P, Clerico M, Cocco E, Conte A, Marfia GA, et al. Disease-Modifying Symptomatic Treatment (DMST) Potential of Cannabinoids in Patients with Multiple Sclerosis. Current Neuropharmacology. 2025; 23: 503–510. https://doi.org/10.2174/011570159X329058240820070701

[2] Fernández Fernández Ó, Costa-Frossard L, Martínez Ginés ML, Montero Escribano P, Prieto González JM, Ramió-Torrentà L, et al. Practical tool to identify Spasticity-Plus Syndrome amongst patients with multiple sclerosis. Algorithm development based on a conjoint analysis. Frontiers in Neurology. 2024; 15: 1371644. https://doi.org/10.3389/fneur.2024.1371644

[3] Goicochea Briceño H, Higueras Ruiz Pérez I, García Domínguez JM, Cuello JP, Medaína Rivera A, et al. Spasticity-Plus syndrome in multiple sclerosis patients in a tertiary hospital in Spain. Frontiers in Neurology. 2024; 15: 1360032. https://doi.org/10.3389/fneur.2024.1360032

[4] Alami Marrouni K, Duquette P. Clinical insights on the spasticity-plus syndrome in multiple sclerosis. Frontiers in Neurology. 2022; 13: 958665. https://doi.org/10.3389/fneur.2022.958665

[5] Patti F, Chisari CG, Fernández Ó, Sarroca J, Ferrer-Picón E, Hernández Vicente F, et al. A real-world evidence study of nabiximols in multiple sclerosis patients with resistant spasticity: Analysis in relation to the newly described ‘spasticity-plus syndrome’. European Journal of Neurology. 2022; 29: 2744–2753. https://doi.org/10.1111/ene.15412

[6] Chan A, Silván CV. Evidence-based management of multiple sclerosis spasticity with nabiximols oromucosal spray in clinical practice: a 10-year recap. Neurodegenerative Disease Management. 2022; 12: 141–154. https://doi.org/10.2217/nmt-2022-0002

[7] Bruno A, Dolcetti E, Centonze D. Theoretical and Therapeutic Implications of the Spasticity-Plus Syndrome Model in Multiple Sclerosis. Frontiers in Neurology. 2022; 12: 802918. https://doi.org/10.3389/fneur.2021.802918

[8] Fernandez O, Costa-Frossard L, Martínez-Ginés ML, Montero P, Prieto-González JM, Ramió-Torrentà L. Integrated Management of Multiple Sclerosis Spasticity and Associated Symptoms Using the Spasticity-Plus Syndrome Concept: Results of a Structured Specialists’ Discussion Using the Workmat® Methodology. Frontiers in Neurology. 2021; 12: 722801. https://doi.org/10.3389/fneur.2021.722801

[9] Fernández Ó, Costa-Frossard L, Martínez-Ginés M, Montero P, Prieto JM, Ramió L. The Broad Concept of “Spasticity-Plus Syndrome” in Multiple Sclerosis: A Possible New Concept in the Management of Multiple Sclerosis Symptoms. Frontiers in Neurology. 2020; 11: 152. https://doi.org/10.3389/fneur.2020.00152

[10] Agencia Española de Medicamentos y Productos Sanitarios (AEMPS). Ficha técnica: cannabinoides en espray (solución para pulverización bucal). Disponible en: https://cima.aemps.es/cima/pdfs/es/ft/72544/FT_72544.html.pdf (Accedido: 25 Abril 2025).
